# Supplementary material for: Silicon-Based On-Chip Tunable High-Q-Factor and Low-Power Fano Resonators with Graphene Nanoheaters
Source: Nanomaterials (Basel). 2023 May 13;13(10):1636. doi: 10.3390/nano13101636 (PMC10222272; doi:10.3390/nano13101636)
Supplement: Supplementary file 1 [file nanomaterials-13-01636-s001.zip › nanomaterials-2343726-supplementary.pdf]

Supporting information for "Silicon-based on chip tunable high Q-factor and low-power Fano resonators with graphene nanoheaters"

Supplementary Note S1: Derivation of the transmittance of the proposed Fano resonator

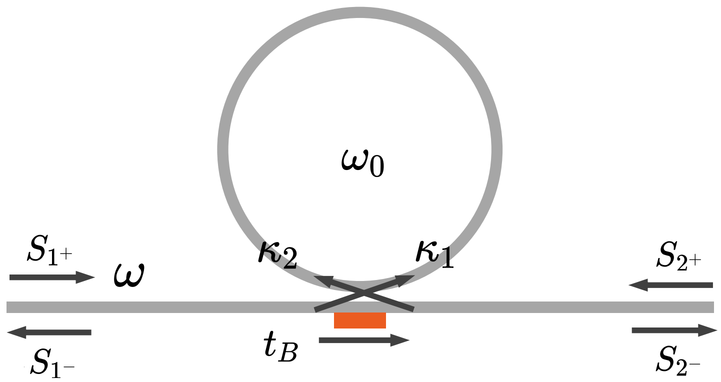

Supplementary Figure S1: The model of the proposed Fano resonator.

To obtain an analytical expression of the transmittance of the proposed structure, the temporal coupled-mode equations is adopted.[1,2] For a two-port single-resonator structure, the intracavity field amplitude $a(\omega, t)$ could be given by

$$\frac{da}{dt} = (-i\omega_0 - \gamma_1 - \gamma_2 - \gamma_A)\, a + \kappa_1 S_{1+} + \kappa_2 S_{2+} \tag{1}$$

where ω is the incident frequency, ω_0 is the resonant frequency of the cavity, $\gamma_{1/2}$ are the decay rates towards the waveguide, γ_A is the intrinsic cavity loss rate, $S_{1+/2+}$ are the amplitudes of the incoming waves from the two ports, and $\kappa_{1/2}$ are the complex coupling coefficients between the ports and the cavity. The outgoing waves towards the two ports are written as

$$\begin{bmatrix} s_{1-} \\ s_{2-} \end{bmatrix} = \boldsymbol{C} \begin{bmatrix} s_{1+} \\ s_{2+} \end{bmatrix} + \begin{bmatrix} \kappa_1 \\ \kappa_2 \end{bmatrix} a, \quad \boldsymbol{C} = \begin{bmatrix} r_B & t_B \\ t_B & r_B \end{bmatrix}. \tag{2}$$

Herein, the scattering matrix $\boldsymbol{C}$ represents the direct coupling between incoming and outgoing waves, with t_B and r_B being the corresponding transmission and reflection coefficients of the amplitudes. And because of the energy conservation, they should satisfy

$|r_B|^2 + |t_B|^2 = 1$, $r_B^* t_B + r_B t_B^* = 0$. Futhermore, time-reversal symmetry requires that $\kappa_j = \sqrt{2\gamma_j} e^{i\theta_j}$ (j $= 1, 2$) and that

$$\boldsymbol{C} \begin{bmatrix} \kappa_1^* \\ \kappa_2^* \end{bmatrix} = - \begin{bmatrix} \kappa_1 \\ \kappa_2 \end{bmatrix}. \tag{3}$$

Based on the equations (1)-(3), the transmittance of the system could be derived as

$$T = \left| \frac{S_{2^-}}{S_{1^+}} \right|^2 = \left| t_B - \frac{2 \left(r_B \sqrt{\gamma_1 \gamma_2} e^{i(\theta_2 - \theta_1)} + t_B \gamma_2 \right)}{i \left(\omega_0 - \omega \right) + \gamma_1 + \gamma_2 + \gamma_A} \right|^2 \tag{4}$$

Supplementary Note S2: COMSOL Multiphysics simula-tions

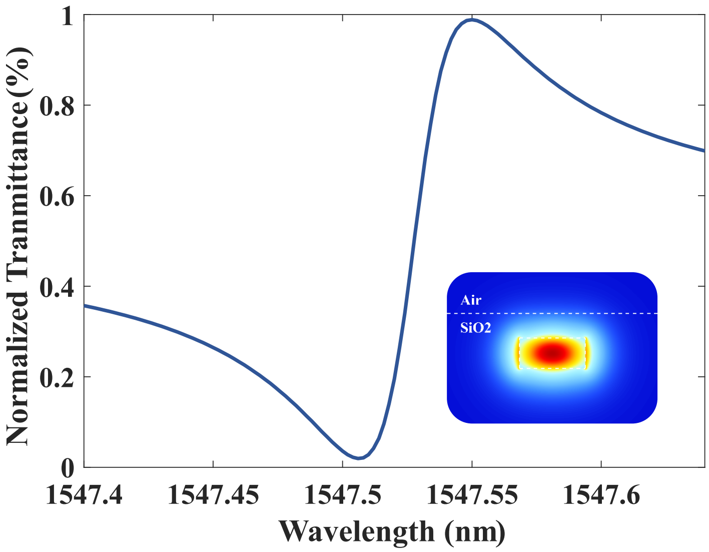

Supplementary Figure S2: The simulated transmittance of Fano resonators. The inset is the distribution of the electric field in the waveguide.

The numerical simulations are conducted using a fully three-dimensional finite element technique (in COMSOL Multiphysics). In simulations, the electromagnetic wave frequency domain (EWFD) module was chosen as the physics field and the first-order scattering boundary as the boundary condition. The boundary mode analysis for both numeric ports was used to find the waveguide propagation mode. The maximal computational mesh size was set to 75 nm, which is around one-sixth of the incident wavelength. A perfect magnetic conductor in an x-y plane was added to reduce the solved number of degrees of freedom. Still, the simulation process requires an intensive computational amount and is time-consuming. For the geometry parameters, the gap of the coupling region is 120 nm, microring radius $R = 16$ μm, and the cross-section of waveguides is 500 nm$\times$220 nm. The length and width of the rectangle block are 1400 nm and 220 nm, respectively.

Supplementary Note S3: Raman spectroscopy and lattice temperature measurements

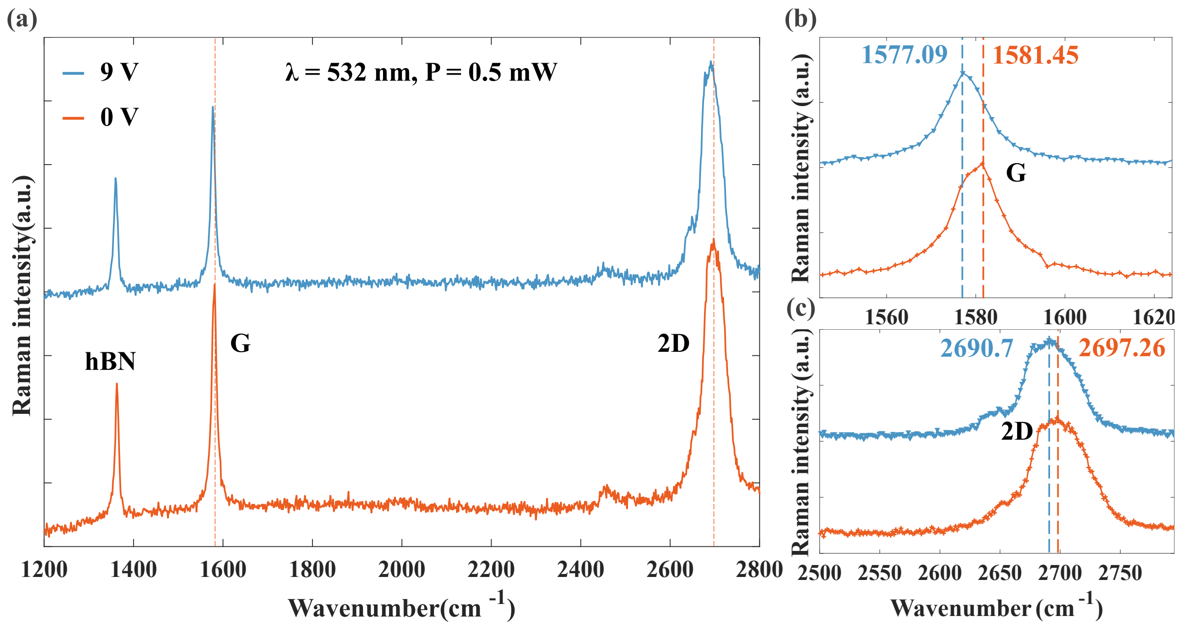

Supplementary Figure S3: (a) Raman spectroscopy of a bilayer graphene covered with hBN at the bias of 0 V and 9 V. Expanded views for the (b) G peak and (c) 2D peak.

The fact that the positions of two distinct Raman-active peaks in bilayer graphene-the G peak at 1581.45 cm^{-1} and the 2D peak at 2697.26 cm^{-1}-are known to undergo a frequency downshift with increasing lattice temperature due to anharmonic phonon coupling. However, since the shift of G peak is influenced by many factors, including strain and doping, 2D peak has been widely employed as a reliable nanometrology of graphene lattice temperature.[3,4] The relation is

$$\omega = \omega_0 + \chi T, \tag{5}$$

where ω_0 is the frequency of 2D mode when temperature T is extrapolated to 0K and χ is the first-order temperature coefficient, which defines the slope of the dependence. Here, χ = -0.066 cm^{-1}/K for bilayer graphene and χ = -0.034 cm^{-1}/K for monolayer graphene, respectively.[3]

Supplementary Note S4: Polycarbonate pick-up methods

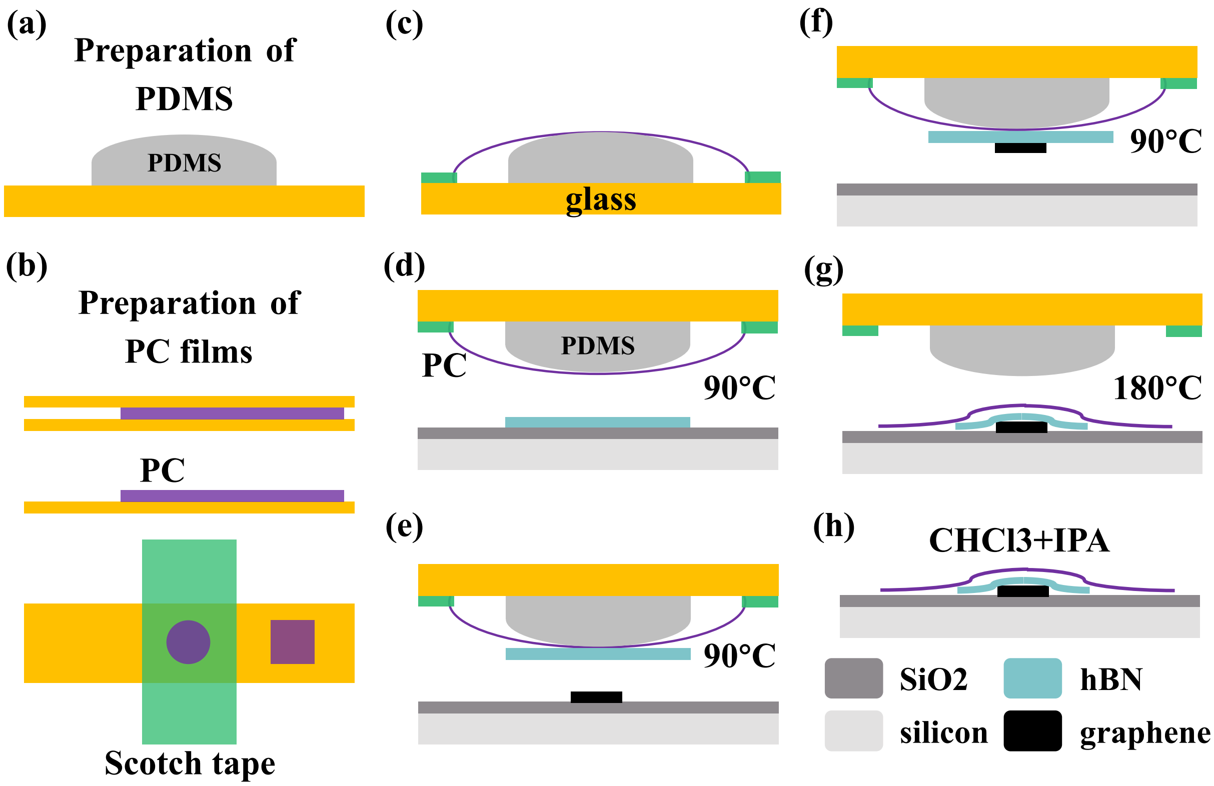

Supplementary Figure S4: (a) PDMS on a glass slide. (b) Preparation of PC films on the glass slide with PDMS. (c) A finished fix-point-transfer glass slide. (d)-(h) Pick-up process of a hBN-graphene stack. Pick up 2D materials at 90°C and release at 180°C.

In the hBN-graphene heterostructure transfer process, polydimethylsiloxane (PDMS) is the support layer on the glass slide, and polycarbonate (PC) film is the colloidal adsorption material during transfer. To make a good glass slide for transferring, we need to prepare PDMS and PC. The following steps are taken to prepare PDMS:

[1] Combine the primary and curing agents in a 10:1 weight ratio.

[2] Stir the prepared solution thoroughly, resulting in numerous bubbles.

[3] Place the solution in a vacuum box until the bubbles disappear.

[4] Drop the prepared solution on the glass slide and heat it for 20 minutes at 125°C to completely solidify, as shown in Figure S4a.

For preparing PC films, the following steps are taken:

[1] Blend PC particles with $CHCl_3$ per the weight proportion of 6:94.

[2] Stir the prepared solution completely at 75°C for 24h through a magnetic stirrer.

[3] Drop 2-3 drops of PC solution on the glass slide, crush another glass slide to straighten the arrangement, and afterward separate the two glass slides equally (Figure S4b).

[4] Exposing them in the air for about 1 minute to shape the PC films.

[5] Use a blade to draw a square of about 1 cm×1 cm on the glass slide after film formation.

[6] Use white tapes to stick away the surrounding film, leaving the target square film.

To combine the PC film with PDMS:

[1] Utilize the puncher to poke a circular opening in the white tape.

[2] Adjust it to the square film and immediately interface it after complete fitting.

[3] Adjust the PC film with the circular opening on the tape to PDMS and attempt to shape it at one time to get the ideal design in Figure S4c.

At length, the preparation of a fix-point-transfer glass slide is finished.

An optical microscope and a motor-driven lifting platform, which can lift objects with a graduation of 1 um, are used to make the stack. After selecting hBN flakes with a thickness of 30-40 nm, we place the samples on the stage, heat them to 90°C, and use transfer slides to pick them up. Then, the bilayer graphene is chosen, aligned with the hBN on the glass slide, and picked up at 90°C. Then the hBN/BLG on the silicon photonics platform is released by heating it to 180°C. After that, we dissolve the PC in chloroform ($CHCl_3$) for five minutes and then wash with isopropanol (IPA), and dry it with nitrogen (Figure S4d-h). The prepared samples are also annealed for three hours at 300°C at high temperature and high vacuum to remove the remaining PC further.

Supplementary Note S5: Experiment measurement

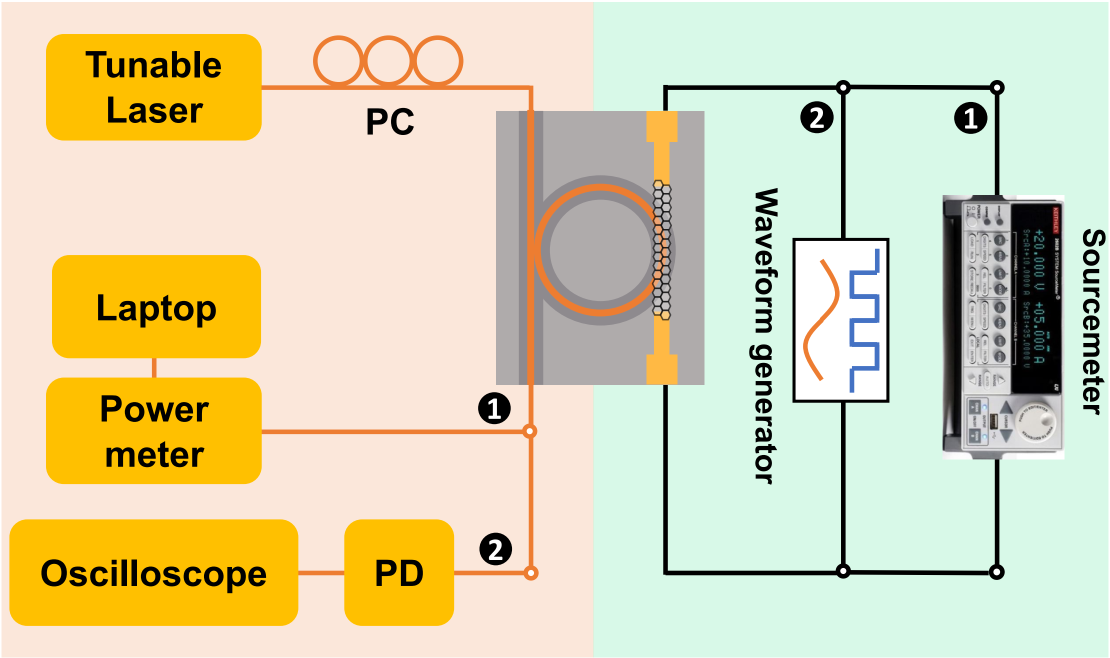

Supplementary Figure S5: A diagram of the experiment setup.

References

(1) Fan, S. Sharp asymmetric line shapes in side-coupled waveguide-cavity systems. *Applied Physics Letters* **2002**, *80*, 908–910

.

(2) Fan, S.; Suh, W.; Joannopoulos, J. D. Temporal coupled-mode theory for the Fano resonance in optical resonators. *JOSA A* **2003**, *20*, 569–572

.

(3) Calizo, I.; Balandin, A.; Bao, W.; Miao, F.; Lau, C. Temperature dependence of the Raman spectra of graphene and graphene multilayers. *Nano Letters* **2007**, *7*, 2645–2649

.

(4) Neumann, C.; Reichardt, S.; Venezuela, P.; Drögeler, M.; Banszerus, L.; Schmitz, M.; Watanabe, K.; Taniguchi, T.; Mauri, F.; Beschoten, B., et al. Raman spectroscopy as probe of nanometre-scale strain variations in graphene. *Nature Communications* **2015**, *6*, 1–7

.
